# The (ANTI)psychotic paradox: Lewy body dementia

**DOI:** 10.1192/j.eurpsy.2021.1106

**Published:** 2021-08-13

**Authors:** J. Galvañ, I. Angélico

**Affiliations:** 1 Psychiatry, Hospital Universitario de La Princesa, Madrid, Spain; 2 Psychiatry, Hospital Universitario Son Espases, Palma de Mallorca, Spain

**Keywords:** psychosis, antipsychotic, dementia, Lewy

## Abstract

**Introduction:**

Lewy Bodie Dementia (LBD) is the second more common progressive dementia caused by the deposition of proteins at the neocortical level, producing motor and psychotic symptoms (parkinsonism and visual hallucinations) which typically get worse with antipsychotics.

**Objectives:**

Find the best antipsychotic treatment in a real patient with LBD balancing control of motor and psychotic symptoms.

**Methods:**

A clinical trial about a real case based on an updated bibliographical review. Received a 70 years old man with more than ten years LBD diagnosis, treated with clozapine (25mg / 12h). According to his wife (principal keeper), it stills a paranoid speech with fluctuant delusional ideas conditioned by visual hallucinations, predominantly in the evening, with no amelioration in four years clozapine treatment, adding a progressive parkinsonism impairment despite neurological drugs (carbidopa:levodopa). Doing a bibliographical review, we found a 2019 article (with 3 Systematic review/Metanalysis and 3 Clinical Practice Guidance, including in NICE), where point olanzapine 5mg well effective but worse tolerated and light up quetiapine as choice that should be considered (no doses specified).

**Results:**

One month later of therapeutic trial following the review in our clinical case, changing clozapine for quetiapine (50mg / 12h), we found an improvement of motor control and a reduction of psychotic manifestation that allows a less disruptive behavior in our patient, also objectified by his principal keeper.

**Conclusions:**

While bibliography doesn’t point a specific dose drug guide for antipsychotic treatment in LBD, in our clinical trial we detected a better control of symptoms using low dose quetiapine, nevertheless more studies are needed.

